# Effect of adding nano-materials on the properties of hydroxypropyl methylcellulose (HPMC) edible films

**DOI:** 10.1038/s41598-023-32218-y

**Published:** 2023-03-28

**Authors:** El-Sayed Khater, Adel Bahnasawy, Basma Abu Gabal, Wael Abbas, Osama Morsy

**Affiliations:** 1 Agricultural and Biosystems Engineering Department, Faculty of Agriculture, Benha University, P.O. Box 13736, Moshtohor, Toukh, Kalubia Egypt; 2 Basic and Applied Science Department, College of Engineering and Technology, Arab Academy for Science and Technology and Maritime Transport (AASTMT), P.O. Box 2033, Cairo, Egypt

**Keywords:** Ecology, Environmental sciences, Engineering

## Abstract

The bio-composite films based on Hydroxypropyl methylcellulose (HPMC) reinforced with silver nanoparticles (AgNPs) and Titanium oxide nanoparticles (TiO_2_-NPs) were developed. Some physical and mechanical properties: Tensile strength (TS), elongation (E), Young’s elastic modulus (EM), water vapor permeability (WVP) and transparency were determined. Antibacterial properties of these films were also studied. The tensile strength values of HPMC film reinforced with Ag NPs and TiO_2_-NPs and HPMC without nanoparticles were 39.24, 143.87 and 157.92 MPa, respectively. Elongation of the HMPC film was less than the HPMC film reinforced with AgNPs and TiO_2_-NPs, the results were 2, 35 and 42%, respectively. Additionally, Young’s elastic modulus of HMPC film was determined to be 19.62 MPa and the HPMC film reinforced with AgNPs and TiO_2_-NPs were 4.11 and 3.76 MPa, respectively. The values of WVP of HMPC film was higher than the HMPC film reinforced with AgNPs and TiO_2_-NPs, where they were 0.5076 × 10^−3^, 0.4596 × 10^−3^ and 0.4504 × 10^−3^ (g/msPa), respectively. Nano-composite films demonstrated strong antibacterial activity against tested pathogen bacteria in the contact surface zone. The antibacterial activites of AgNPs (~ 10 nm) at 80 ppm were more active than 20 and 40 ppm against foodborne pathogen i.e. *Bacillus cereus* and *Escherichia coli*, the inhibition zone diameters were 9 and 10 mm, respectively. As well, TiO_2_-NPs (~ 50 nm) at 80 ppm were more active than 20 and 40 ppm against *B. cereus* and *Salmonella Typhimurium*, the inhibition zone diameters were11 and 10 mm, respectively.

## Introduction

In food sector, using nano-materials became very important and attractive, specially packing materials. Edible films and coating materials are commonly used as appropriate package materials to prolong the shelf-life of fresh food. These nanomaterials have distinguished properties compared to other materials due to their high surface area to volume ratio and other unique physiochemical properties such as color, solubility, strength, diffusivity, toxicity, magnetic, optical, and thermodynamic, etc.^1^. Nanotechnology has brought new industrial revolution and both developed and developing countries are interested in investing more in this technology^2^. Therefore, nanotechnology offers a wide range of opportunities for the development and application of structures, materials, or system with new properties in various areas like agriculture, food, and medicine, etc. The marketing of nanofood was estimated about US $35.5 billion in 2013 and US $100 billion in 2020^3^.

Cellulose is the most abundant organic compound in the environment, which is renewable, recyclable, and biodegradable (into carbon, hydrogen, and oxygen)^4^. Notably, cellulose is more suitable for packing purpose as it is not a thermoplastic polymer, whereas its ester derivatives (methylcellulose (MC), hydroxypropyl methylcellulose (HPMC), hydroxypropyl cellulose (HPC), and ethyl cellulose (EC)) are biodegradable thermoplastic polymers. Hydroxypropyl methylcellulose and MC are soluble in the cold water, but after heating they form a thermally reversible and relatively hard gel by heating process at 50–80 °C^5,6^. Hydroxypropyl methylcellulose is odorless, flavorless, transparent, stable, oil‐resistant, nontoxic, and edible material with good film‐forming properties. It is a nonionic polymer with a linear structure of glucose molecules, in which its matrix is stabilized using hydrogen bonds^7,8^.

Silver nanoparticles are among the most explored nanoparticles, owing to their established antimicrobial potential against multiple commensals and pathogenic strains^9^. Besides bacterial strains, silver nanoparticles are known to be inhibitory against multiple fungi and also several viruses^10^. Silver targets bacterial metabolism by binding to its DNA, proteins and enzymes; resulting into bacteriostatic effects^11^. Silver nanoparticles destabilize and disrupt both the outer and cytoplasmic membranes^12^. Silver nanoparticles also inhibit the respiratory chain enzymes and can also stimulate the production of reactive oxygen species (ROS)^13^.

Naturally, titanium dioxide exists in three primary phases i.e., anatase, rutile, and brookite; having varied crystal sizes (diameter ranged from 2 to 6 nm)^14^. TiO_2_ possess photocatalytic abilities and at nanoscale TiO_2_ shows surface reactivity, which connects it with biological molecules (phosphorylated proteins and peptides) and DNA^15^. The surface energy of TiO_2_ nanoparticles amplifies with size the antibacterial properties of TiO_2_ is well known^16^ however, the antibacterial capacity of nano-TiO_2_ particles confined to the exposure of UV irradiation^17^. Although, the exact mechanism of biocidal activity of TiO_2_ is unclear, it may be attributed to its initial oxidative attack over the outer/inner bacterial cell membrane, alterations of TiO_2_ is unclear, it may be attributed to its initial oxidative attack over the outer/inner bacterial cell membrane, alterations of Coenzyme A-dependent enzyme activity, and DNA damage through hydroxyl radicals^18^.

Tensile strength (TS), water vapor permeability (WVP), % elongation (E), adsorption capacity and % soluble matter (SM) of water were studied in hydroxypropyl methylcellulose (HPMC) films reinforced by polyethylene glycol (PEG). The TS between 17 and 44 N/mm^2^ and the WVP of films was determined to be 0.232·10^10^–1.160·10^10^ g/msPa and %E between 14 and 97%, depending on composition. Moisture content (MC), ethanol content and pressure of PEG affected the film formation. Adding PEGs to the polymer matrix increased the WVP, elongation (E) and solubility but decreased the tensile strength (TS)^19^.

Food safety is an important concept and health concern in developed and developing countries^20^. The Center for Disease Control and Prevention Center^21^ reported that about 179 million people get sick, 428,000 hospitalized, 6000 deaths, and costed US $15.6 billion every year in USA from five food-borne pathogens. In addition, the World Bank report^22^ the food-borne illness in developing countries costed ~ US $110 billion, 600 million illness cases, and 420,000 premature deaths in Asia and Africa. Five-food-borne pathogens record about (88%) of the listed food concerned deaths: *Salmonella nontyphoidal* (35%), *Norovirus* (26%), *Campylobacter* (15%), *Toxoplasma gondii* (8%), and *Escherichia coli* (4%)^21^. One way to control food-borne pathogens and food spoilage is to develop antimicrobial films for packaging food.

Studying the properties of these film is very important for food preservation, therefor, the main aim of this work is to develop an edible film that made from hydroxypropyl methylcellulose (HPMC) reinforced with nanoparticles (AgNPs and TiO_2_NPs) and study some mechanical and antibacterial properties of these films. These properties include: tensile strength (TS), elongation (*E*), Young`s elastic modulus (EM), water vapor permeability (WVP) and transparency.

## Materials and methods

### Materials

Silver nanoparticles (AgNPs), Titanium oxide nanoparticles (TiO_2_-NPs) and glycerol were purchased from Nano Gate Company, Cairo, Egypt. Hydroxypropyl methyl cellulose (HPMC) was supplied from G.I.D.C industrial Estate, India. *Bacillus cereus* (ATCC7464), *Salmonella Typhimurium* (ATCC14028), *E. coli* (ATCC87939), and *Staphylococcus aureus* (ATCC 6538) were obtained from (Microbiological Resources Center, MIRCEN, and Cairo, Egypt). As shown in Table 1, the compounds of bio-composite films were made from Distilled water, HMPC, glycerol, AgNPs and TiO_2_NPs.

**Table 1:** Constitutes of HMPC films reinforced with nanoparticles.

Films	Distilled water (ml)	HMPC (g)	Glycerol 30% (ml)	AgNPs (ppm)	TiO_2_NPs (ppm)
HMPC-Control	1000	40	10	–	–
HMPC-AgNPs	1000	40	10	80	–
HMPCTiO_2_NPs	1000	40	10	–	80

*HPMC* hydroxyl propyl methyl cellulose film, *HMPC-AgNPs* hydroxyl propyl methyl cellulose films reinforced with silver Nanoparticles, and *HMPC-TiO*_*2*_* NPs* hydroxyl propyl methyl cellulose films reinforced with titanium oxide nanoparticles.

### Nanoparticles preparation

Silver nanoparticles (AgNPs) have been prepared by chemical reduction method as reported by Pal et al^23^. For the synthesis of Ag nanoparticles SHARP make microwave oven (model: R259) was used. In a typical procedure, 10 ml of 1% (w/v) ethanolic solution of polyvinyl pyrrolidone (PVP) and 0.2 ml of 0.1 M AgNO_3_ were taken in a 25 ml closed conical flask and placed in a microwave oven that was operated at the 100% power of 800 W and frequency 2450 MHz for 5 s. The colorless solution instantaneously turned to the characteristic pale yellow colour, indicating the formation of silver nanoparticles. The advantage of microwave-mediated synthesis over the conventional heating is the improved kinetics of the reaction generally by one or two order of magnitude, due to rapid initial heating and the generation of localized high-temperature zones at reaction sites^24^.

Titanium oxide nanoparticles (TiO_2_-NPs) were prepared according to Salah et al^25^ with some modification. Briefly, the metallic compounds size (~ 0.6 ± 1 μm, 120 purity 99.9%, Loba, Chemi, Pvt. Ltd, India) was milled in steel cells (250 mL) using hardened steel balls (diameter 15 mm, weight 32 g) in ambient atmosphere for different times ranging from 2 to 50 h. The mechanical milling was performed in a horizontal oscillatory mill (Retsch, PM 400) operating at 25 Hz. The mixture ratio of steel balls and powders was around 15:1 by weight percent. Two parallel cells were used in this experiment (the total weight for the sample powder was 20 g).

### Preparation of bio-composite films based on hydroxypropyl methyl cellulose (HPMC) reinforced with nanoparticles

**Figure 1:**
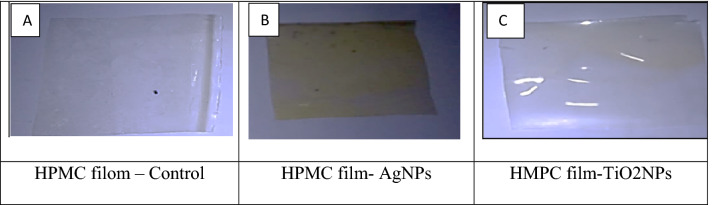
HPMC film.

Hydroxy propyl methyl cellulose (HPMC) was prepared according to De Moura et al^26^ with some modification. Briefly, each 40 g HPMC was dissolved in 1000 mL distilled water at 70 °C and stirred at 1000 rpm/min for 2 h to a complete dissolving. A 1 mL of glycerol 30% was add as plasticizer, then 80 ppm of different nanoparticles were added with stirring for 30 min. The solution was autoclaved (121 °C/15 min at 15 psi). Then casted onto glass petri dishes 25 × 20 cm in sterilized condition and allowed to dry overnight (18 h) in a laminar air flow at 25 °C and kept under cold storage until used. In Fig. 1a. HPMC control film without nanoparticles, Fig. 1b. HPMC film reinforced with silver Nanoparticles concentration at 80 ppm and Fig. 1c. HPMC film reinforced with titanium oxide nanoparticles concentration at 80 ppm.

### Characterization of Ag nanoparticles (AgNPs)

Characteristic optical properties of Ag nanoparticles were recorded using PerkinElmer Lambda 35 UV–vis spectrophotometer. Spectra were recorded using 1 cm^3^ quartz cell. Emission spectrum (220 nm) of the solution was recorded by using spectrofluorometer from JASCO. Size, shape and particle size distributions were determined using a JEOL JEM-2011 transmission electron microscope operated at an accelerating voltage of 200 kV. Images were recorded using a Gatan DualVision 600t CCD camera attached to the microscope and were analyzed using Gatan Digital Micrograph Version 3.11.1. The TEM was calibrated for diffraction and imaging mode using standard samples. The resolution of the system was calibrated with manganese (Mn)^27^. Samples were prepared for TEM analysis by placing a drop of the solution on a carbon coated copper grid and drying in air. The energy dispersive X-ray analysis was undertaken with a Princeton Gamma Tech Prism 1G system with a 10 mm^2^ silicon detector attached to the TEM and the peaks were analysed with Imix 10.594 software^27^.

### Characterization of titanium oxide nanoparticles (TiO_2_-NPs)

The X-ray diffraction (XRD) of nanoparticles were measured using X-ray diffractometer (Rigaku D/Max-B, Tokyo, Japan**)** according to Akbari et al^28^. The samples were put onto glass slide and the spectra were recorded using Cu radiation (wavelength of 0.1541 nm) and a nickel monochromator filtering wave at 40 kV and 30 mA. The average crystallite size of (TiO_2_-NPs) was estimated using Scherer's equation:

1 $$ D = \frac{0.9\lambda }{{\beta \cos \theta }} $$

where D is the crystallite size, λ is the X-ray wavelength, β is the full width at have maximum of the peak and θ is the central angle of the diffraction peak.

### Antibacterial activities of nanoparticles against foodborne pathogens

Antibacterial activities of nanoparticles against food-borne pathogens was done by disk diffusion method on tryptic soy agar media (TSA) according to Salari et al^29^. The inoculum (100 μl) was adjusted nanoparticles employed in this study (20, 40 and 80 ppm), were measured after incubation at 37 °C for 24–48 h. the zones of bacterial growth inhibition were measured in mm unit.

### Mechanical properties of edible composite films reinforced with nanoparticles

#### Film thickness

Thicknesses of films were measured with a digital micrometer (Mitutoyo Manufacturing Co. Ltd., Japan, sensitivity ± 0.001 mm at 5 random positions on the film, following WVP and preceding tensile tests. WVP and mechanical properties were calculated based on average thickness^19^.

#### Tensile strength (TS), elongation at break (EAB), and Young`s elastic modulus (EM)

The TS, EAB, and EM of composite edible film were determined according to Hazirah et al^30^. An Instron Universal Testing Instrument (Model 1011) was used to determine film TS and %E. Testing film specimens were rectangular strips 38 mm long and 5.79 mm wide as suggested in ASTM D683M^31^. A strain rate of 50 mm/min was used. All film strips were equilibrated for one week to 52 ± 2% RH in a cabinet using saturated magnesium nitrate solution at room temperature (25 ± 1 C). At least four replicates of each MC film were tested. All three tests were performed in edible composite films^31^. Values for TS, EAB, and EM were calculated using:

2 $$ TS({\text{MPa}}) = \frac{{F_{\max } (N)}}{{A(m^{2} )}} $$

where F_max_ is the max load (N) needed to pull the sample apart and A is the cross sectional area m^2^ of the film sample.

3 $$ EAB(\% ) = \frac{{l_{\max } }}{{l_{0} }} \times 100 $$

where l_max_ is the film elongation (mm) at that moment of rupture and l_o_ is the initial grip length (mm) of the sample.

4 $$ EM({\text{MPa}}) = \frac{Stress}{{Strain}} $$

where stress is load (N) divided by area (mm^2^) and strain is change in length (mm) divided by original length (mm).

#### Water vapor or permeability (WVP)

WVP of films was determined gravimetrically at 25 ± 1 °C using a modified ASTM E96-80^31^ procedure. The test film was sealed to a glass dish containing anhydrous calcium chloride (Merck, Darmstadt, Germany), 0% RH, and the dish was placed in a desiccator maintained at 52 ± 2% RH with saturated magnesium nitrate (Merck, Darmstadt, Germany). The water vapor transferred through the film and absorbed by the desiccant was determined by measuring the weight gain. WVP was calculated from the following equation:

5 $$ WVP = C\frac{x}{A\Delta P} $$

where WVP is in g/msPa, x is the film thickness (m), A is area of the exposed film (m^2^), DP is the water vapor pressure differential across the film (Pa), and C is the slope of the weight gain of the dish, to the nearest 0.0001 g, versus time. Generally, ten weighing were taken over a 7–10 h period. Slopes were calculated by linear regression and correlation coefficient (r^2^) for all reported data were 0.99 or greater. At least three replicates of each film type were tested for WVP.

#### Transparency

**Figure 2:**
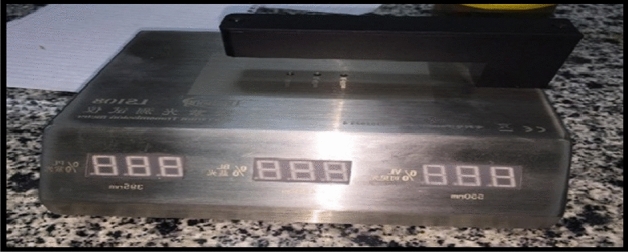
LS108 Spectrum Transmission Meter.

Figure 2 shows Transparency of the films reinforced with nano-materials was determined by LS108 Spectrum Transmission Meter PL transmission meter, BL transmission meter, Light Transmittance Meter according to Hazirah et al^30^. It has following feature and Parameter**:**

Feature:

1. PL transmission meter, BL transmission meter, Light Transmittance Meter, Three function in ONE device.
2. Self-calibration and auto-calibration, NO need any manual adjustments.
3. Simple operation, putting the testing sample in the testing position, PL, BL and VL transmission values of the sample will simultaneously display.

Parameter:

1. Size: 200 mm*180 mm*106 mm (L*W*H)
2. Size of Testing sample: > ￠3 mm
3. Weigh: 1500 g
4. Resolution: 0.1%
5. Accuracy: ± 2% (Colorless and transparent material)
6. Purple light Peak wavelength: 395 nm
7. Blue light Peak wavelength: 460 nm
8. Visible light Peak wavelength: 550 nm
9. Power supply: 5 V DC Adapter.

## Results and discussion

The properties of nano-materials that used in making packaging films such as silver (AgNPs) and titanium oxide (TiO_2_NPs) were studied. The properties of the films such as tensile strength (TS), elongation (*E*), Young`s elastic modulus (EM), water vapor permeability (WVP), transparency and antibacterial properties of bio-composite film were studied for the edible films reinforced with AgNPs and TiO_2_ -NPs.

### Characterization of Ag nanoparticles (AgNPs)

**Figure 3:**
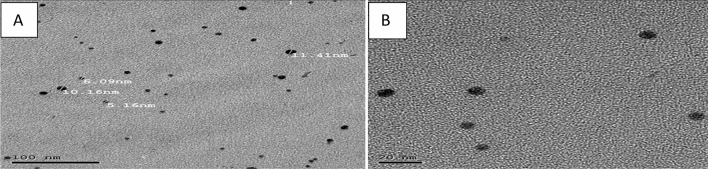
Shows the TEM of AgNPs a different scales (**A**) 100 nm, (**B**) 20 nm.

**Figure 4:**
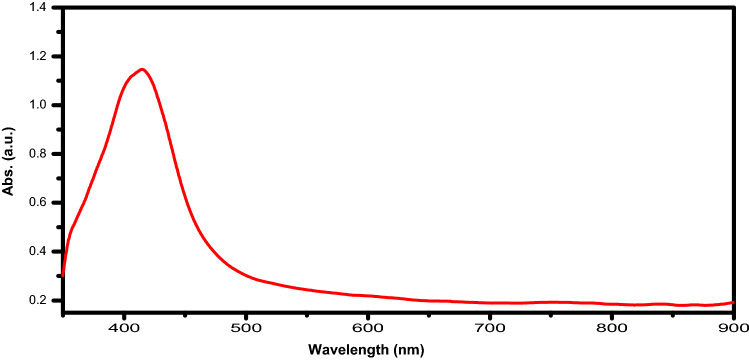
UV–vis and fluorescence spectroscopy of silver nanopartic.

Figure 3 shows that the spherical particles sizes were of 10 ± 2 nm diameter. TEM analysis was carried out on 100 times dilution of colloidal suspension only few particles were observed in the small section of high-resolution image. Utilization of microwave irradiation treatment showing good results not only due to faster heating but it gives uniformly distributed monodispersed particles. Colour of the solution was changed by the formation of silver nanoparticles. The characteristic surface plasmon band at 416 nm that is slightly higher was shown the visible spectrum of silver nanoparticles (Fig. 4). These results agreed with those obtained by Pal et al^23,27^. Silver nanoparticles had refractive index of the surrounding medium because of its slightly red shift on surface plasmon.

### Characterization of titanium oxide nanoparticles (TiO_2_-NPs)

**Figure 5:**
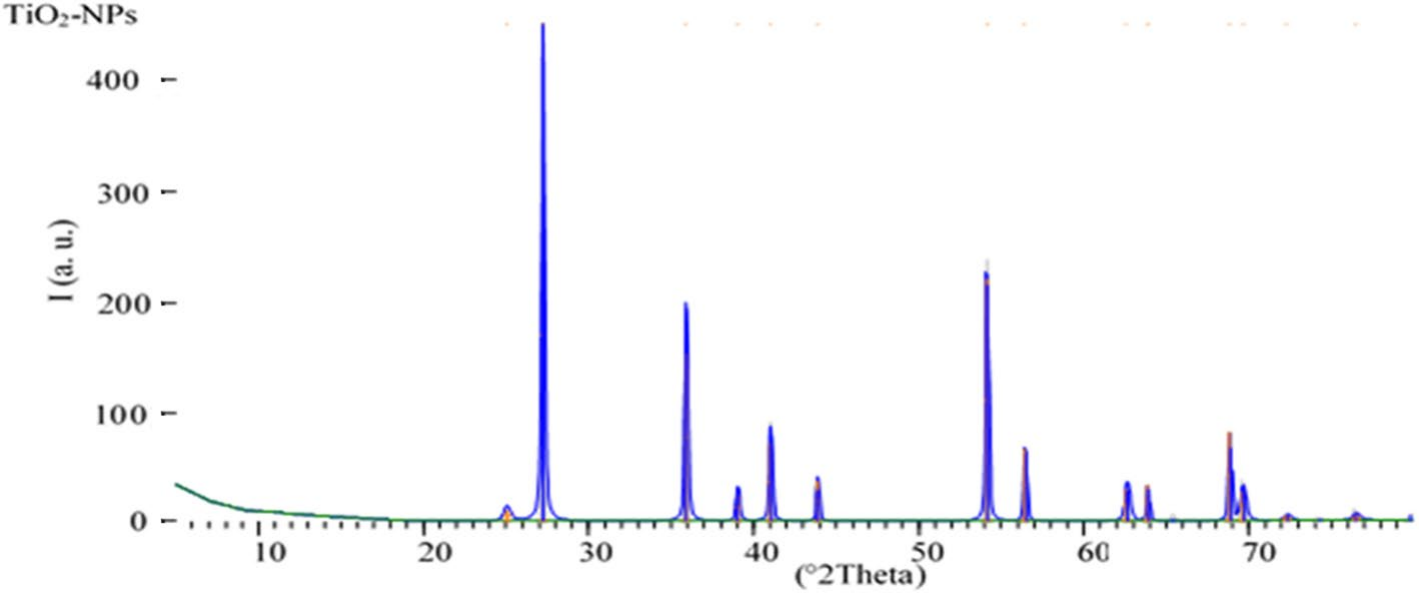
XRD pattern of titanium oxide nanoparticles (TiO_2_-NPs).

TiO_2_-NPs were measured by XRD to determine the crystallite size and the purity of nanoparticles. The crystallinity size of nanoparticles was confirmed by XRD analysis as shown in Fig. 5. The XRD spectrum of dry nanoparticles were high purity, clear and broad peaks. The XRD pattern fits well with a wurtzite structure and the average crystal (diameter). Therefore, the results XRD characterization allow to conclude the nanoparticles size have a radius of around 50 ± 5 nm. These results are accordance with obtained by Aboud et al^32^.

### Antibacterial activities of nanoparticles against foodborne pathogen

Table 2 shows **t**he antibacterial activities of inorganic nanoparticles i.e. Silver nanoparticles (AgNPs) and Titanium oxide nanoparticles (TiO_2_-NPs) against four food-borne pathogens: *Bacillus cereus, Salmonella Typhimurium, E.coli* and *Staphylococcus* aureus were evaluated results conducted that Ag-Nps (~ 10 nm) and TiO_2_-NPs (~ 50 nm) at 80 ppm were effective against food-borne pathogens i.e. *B. cereus, S. Typhimurium, E. coli 0157:H7* and *S. aureus*, than 20 and 40 ppm respectively. These result with partially agreement those result indicated by Khezerlou et al^33^ and Ejaz et al^34^. Moreover, AgNPs at 80 ppm were more effective against *B.Cereus* and *E. Coli* these results agreement with data those reported by Nanda and Saravanan^35^. As well, TiO_2_-NPs at 80 ppm were more active against *B.cereus* and *S. Typhimurium* these results were similar to the results those obtained by Martinez-Gutierrez et al^36^. AgNPs and TiO_2-_ NPs incorporated composite films demonstrated strong antibacterial activity against both the Gram-positive and Gram-negative food borne pathogenic bacteria.

**Table 2:** Antibacterial activities of nanoparticles at different concentration against food-borne pathogens bacteria.

Nano particles agent (ppm)	Bacterial strains Inhibition zone diameter ( mm )											
	*S. Typhimurium*			*E. coli*			*S. aureus*			*B. cereus*		
	20	40	80	20	40	80	20	40	80	20	40	80
Ag-Nps	6 ± 0.1	7 ± 0.1	8 ± 0.04	5 ± 0.01	8 ± 0.1	10 ± 0.2	5 ± 0.1	7 ± 0.3	8 ± 0.1	7 ± 0.1	8 ± 0.2	9 ± 0.2
TiO_2_-Nps	8 ± 0.02	9 ± 0.01	10 ± 0.01	7 ± 0.1	8 ± 0.1	9 ± 0.1	6 ± 0.2	7 ± 0.2	8 ± 0.2	8 ± 0.1	9 ± 0.2	11 ± 0.1

Values were presented as mean ± standard deviation (SD).Ag-NPs: silver Nanoparticles and (TiO_2 -_NPs): titanium oxide nanoparticles.

**Figure 6:**
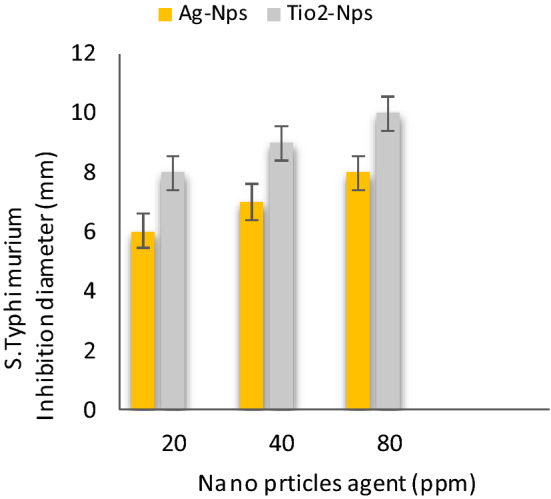
Antibacterial activities of nanoparticles at different concentrations against *S. Typhimurium.*

**Figure 7:**
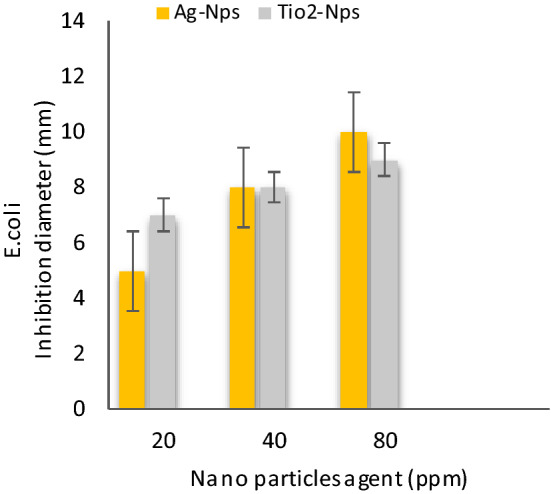
Antibacterial activities of nanoparticles at different concentrations against *E. coli.*

**Figure 8:**
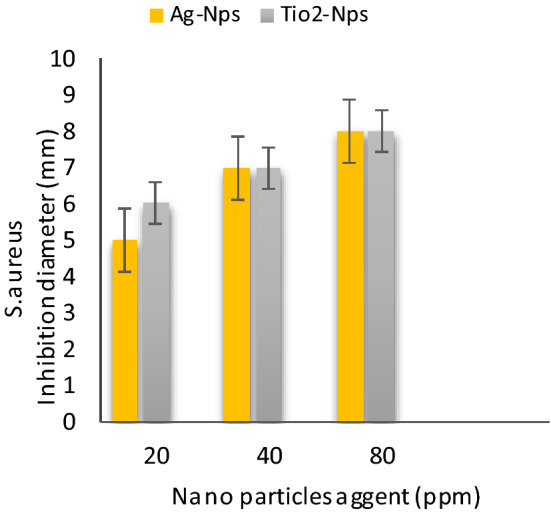
Antibacterial activities of nanoparticles at different concentrations against *S. aureus.*

**Figure 9:**
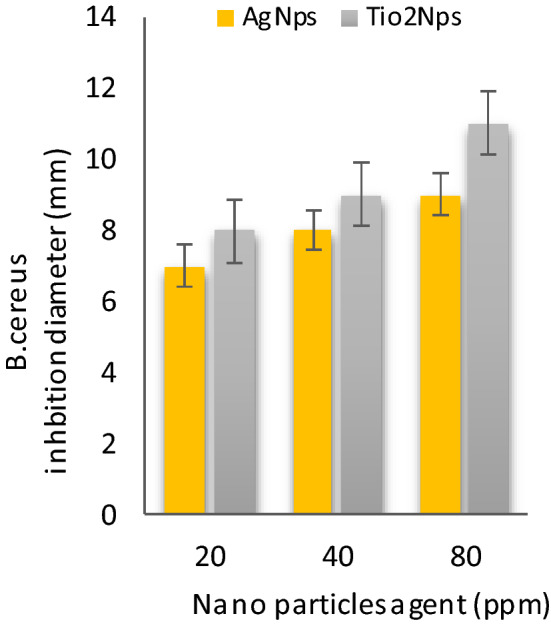
Antibacterial activities of nanoparticles at different concentrations against *B. cereus.*

Figure 6 shows the antibacterial activities of silver nanoparticles (Ag-NPs) and titanium oxide nanoparticles (TiO_2_-NPs) at different concentrations 20, 40 and 80 ppm against *S.Typhimurium.* The results were at a concentration of 80 ppm for (Ag-NPs) and (Tio_2_-NPs) more value than 20 and 40 ppm, the inhibition zone diameter was 8 and 10 mm, respectively, in Fig. 7 shows that the antibacterial activities of silver nanoparticles (Ag-NPs) and titanium oxide nanoparticles (TiO_2_ -NPs) at different concentration 20, 40 and 80 ppm against *E. coli.* The results were at a concentration of 80 ppm for (Ag-NPs) and (Tio_2_ -NPs) more value than 20 and 40 ppm, the inhibition zone diameter was 10 and 9 mm, respectively, in Fig. 8 shows the antibacterial activities of silver nanoparticles (Ag-NPs) and titanium oxide nanoparticles (TiO_2_-NPs) at different concentrations 20, 40 and 80 ppm against *S.aureus.* The results were at a concentration of 80 ppm for (Ag-NPs) and (TiO_2_ -NPs) more value than 20 and 40 ppm, the inhibition zone diameter was 8 and 8 mm, respectively, and in Fig. 9 shows the antibacterial activities of silver nanoparticles (Ag-NPs) and titanium oxide nanoparticles (TiO_2_-NPs) agent at concentrations 20, 40 and 80 ppm against *B.cereus*. The results were at a concentration of 80 ppm for (Ag-NPs) and (TiO_2_-NPs) more value than 20 and 40 ppm, the inhibition zone diameter was 9 and 11 mm, respectively.

### Mechanical properties of HPMC films reinforced with nanoparticles

#### Film thickness

The average of bio-composite films thickness was tested (HMPC, HMPC reinforced with AgNPs, and HMPC reinforced with TiO_2_NPs). As shown in Table 3**,** the results values of control film (HMPC), HMPC-AgNPs, and HMPC-TiO_2_NPs were 0.30, 0.19, and 0.12 µm, respectively.

**Table 3:** Thickness of bio-composite films reinforced with nanoparticles.

Films	Thickness (µm)
HPMC-Control	0.30 ± 0.2
HMPC-AgNPs	0.19 ± 0.02
HMPC-Tio_2_ NPs	0.12 ± 0.01

Values were presented as mean ± standard deviation (SD). Control -HPMC: hydroxyl propyl methyl cellulose, HMPC-AgNPs: HMPC reinforced with silver Nanoparticles, and HMPC-Tio_2 -_NPs: HMPC reinforced with titanium oxide nanoparticles.

#### Tensile strength (TS), elongation at break (EAB%) and Young`s elastic modulus (EM)

The mechanical properties such as tensile strength, elongation, and Young`s elastic modulus were evaluated. As shown in Table 4 the tensile values of HPMC film reinforced with Ag NPs and TiO_2_-NPs were higher than that of tensile strength of HPMC films without nanoparticle (control), the results values were 39.24, 143.87and 157.92 MPa, respectively, for HMPC, HMPC reinforced with AgNPs, and HMPC reinforced with TiO_2_NPs. On the other hands, elongation was tested, the results obtained that, the HPMC film reinforced with Ag NPs and TiO_2_-NPs have higher value of elongation compared to than HPMC films without nanoparticle (control), the results values were 2, 35 and 42%, respectively, for HMPC, HMPC reinforced with AgNPs, and HMPC reinforced with TiO_2_NPs. In addition to, Young`s elastic modulus was evaluated, the results show that, HPMC film reinforced with Ag NPs and TiO_2_-NPs have lower values compared to than HPMC films without nanoparticle (control). The elongation values were 19.62, 4.11 and 3.76 MPa, respectively. That is due to (a) the nanoparticles’ ability to filling pore between HPMC film structures. (b) The water evaporates permeability during film formation (c) Hence, the increased surface area reinforces the (d) film thickness and biodegradable. These results are in agreement with those obtained by Martinez-Gutierrez et al^36^, Jiménez et al^37^, Silva-Weiss et al^38^, Ahmadi et al^39^, Osorio et al^40^ and Sievens-Figueroa et al^41^.

**Table 4:** tensile strength, elongation, and Young`s elastic modulus of bio-composite films reinforced with nanoparticles.

Mechanical properties	HMPC-Control	HMPC-AgNPs	HMPC-TiO_2_NPs
Tensile (MPa)	39.24	143.87	157.92
Elongation (%)	2	35	42
Young ′s elastic modulus (MPa)	19.62	4.11	3.76

Control (HPMC): hydroxyl propyl methyl cellulose film, HMPC-AgNPs: hydroxyl propyl methyl cellulose films reinforced with silver Nanoparticles, and (HMPC-TiO_2_ NPs): hydroxyl propyl methyl cellulose films reinforced with titanium oxide nanoparticles.

**Figure 10:**
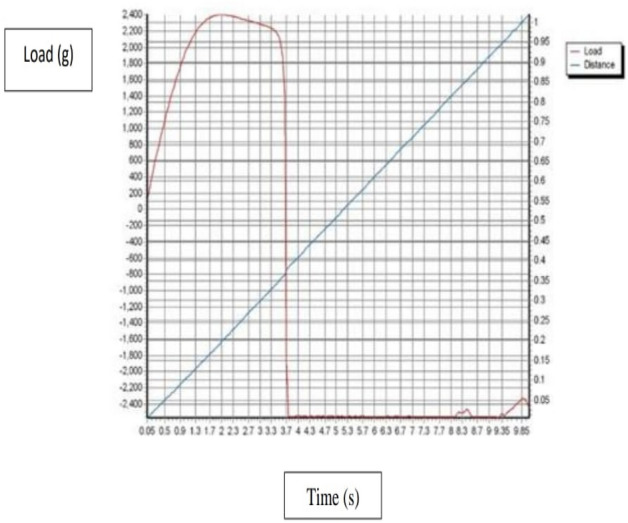
Texture Curve of HPMC film (Control).

**Figure 11:**
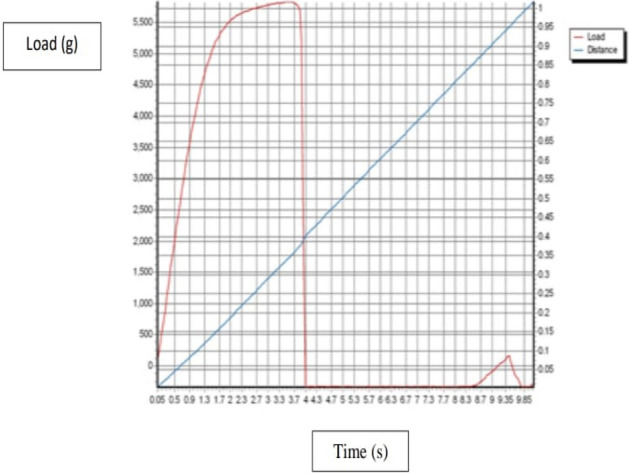
Texture Curve of HPMC film reinforced with AgNPs.

**Figure 12:**
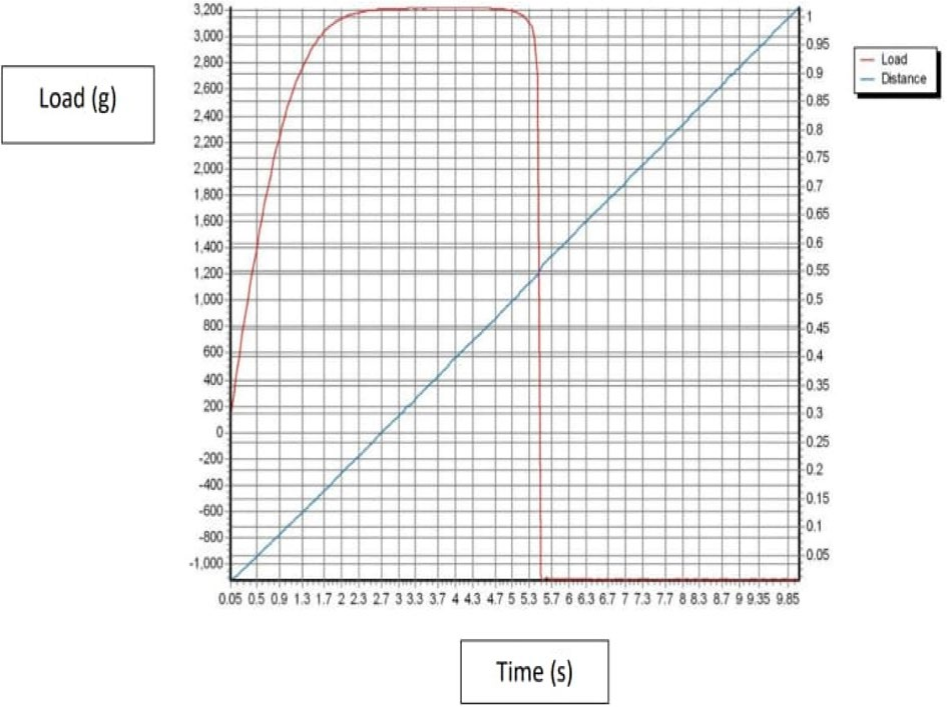
Texture Curve of HPMC film reinforced with TiO_2_-NPs.

The mechanical properties such as tensile, elongation, and Young`s elastic modulus were evaluated by Using a texture analyze. In Fig. 10 shows Texture Curve of HPMC film (Control), Fig. 11 a texture analyze shows Texture Curve of HPMC- AgNPs, and Fig. 12 a texture analyze shows Texture Curve of HPMC- TiO_2_ NPs.

#### Water vapor permeability (WVP)

**Figure 13:**
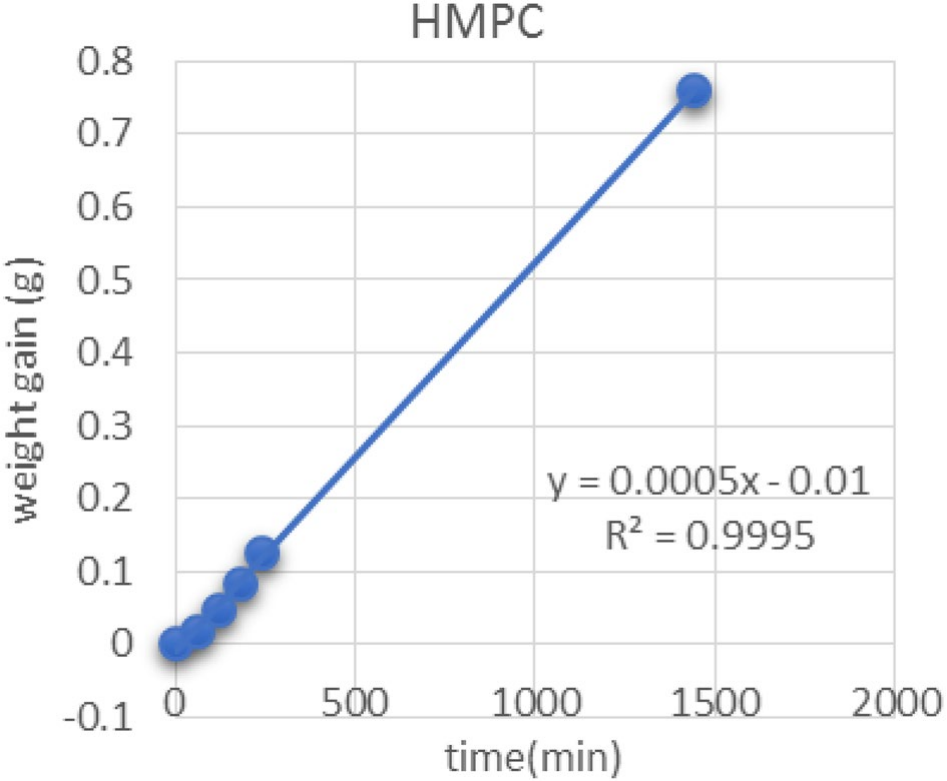
Graph of HMPC film.

**Figure 14:**
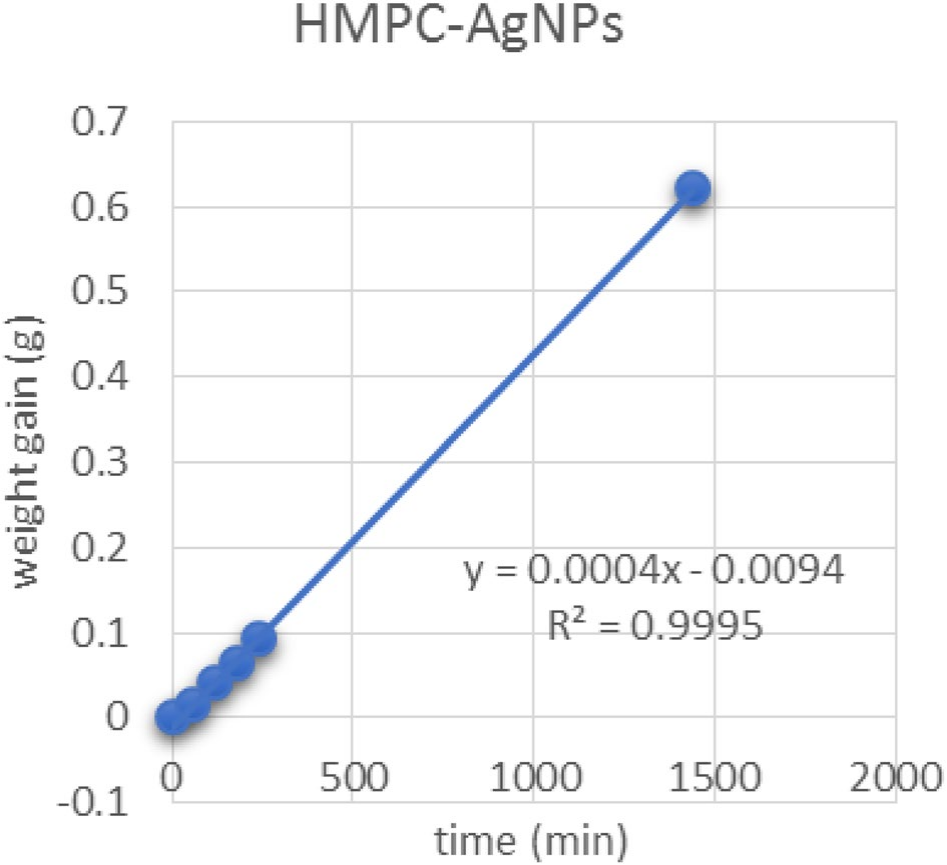
Graph of HMPC film reinforced with Ag NPs.

**Figure 15:**
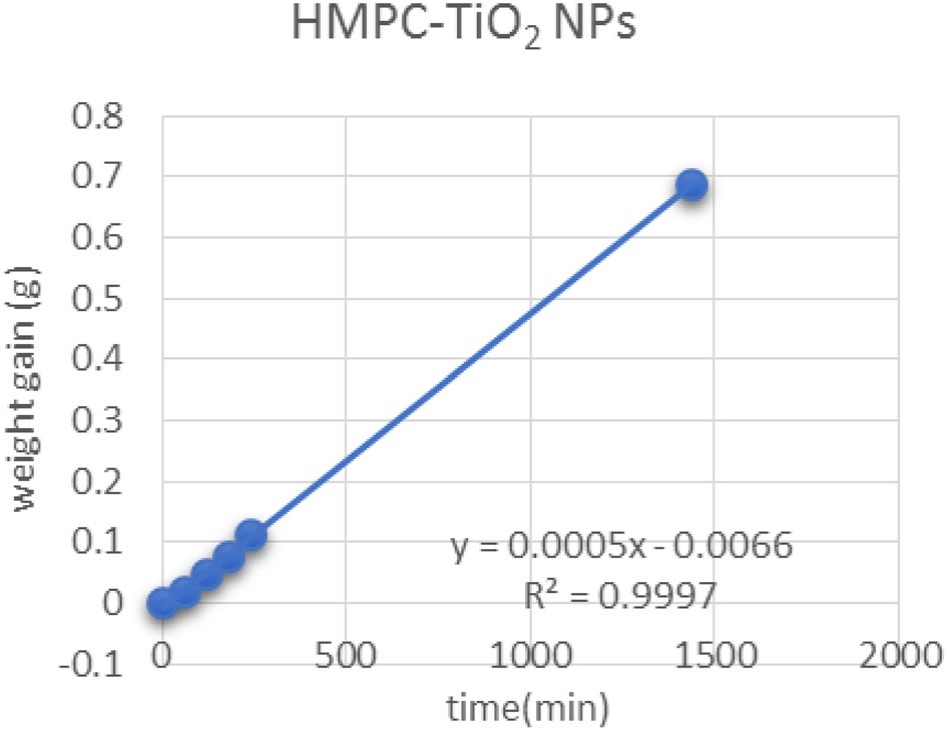
Graph of HMPC film reinforced with TiO_2_ NPs.

Figures 13, 14 and 15 show the relationship between the weight gain and time to calculate the slope (C) by linear regression (Y) and correlation coefficient (r^2^) which is used to determine of WVP transferred through the film was determined by measuring the weight gain. As shown in Table 5, the slope of bio-composite films which is used to determine of water permeability of bio-composite films reinforced with nanoparticles.

**Table 5:** The slope of bio-composite films which is used to determine of water permeability of bio-composite films reinforced with nanoparticles.

Samples	Linear regression equation (Y)	Correlation coefficient(r^2^)	Slope (C)
HMPC-Control	Y = 0.0005x − 0.01	R^2^ = 0.9995	0.0005
HMPC-AgNPs	Y = 0.0004x − 0.0094	R^2^ = 0.9995	0.0004
HMPC-TiO_2_NPs	Y = 0.0005x − 0.0066	R^2^ = 0.9997	0.0005

*HPMC*: hydroxyl propyl methyl cellulose film, *HMPC-AgNPs* hydroxyl propyl methyl cellulose films reinforced with silver Nanoparticles, and *HMPC-TiO*_*2*_* NPs*: hydroxyl propyl methyl cellulose films reinforced with titanium oxide nanoparticles.

Table 6 shows the average of weight gain with time to determine of WVP of bio-composite films. The WVP results showed that, HPMC film reinforced with Ag NPs and TiO_2_-NPs were less value than that of HPMC films without nanoparticle (control), the results values were 0.5076 × 10^–3^ and 0.4596 × 10^–3^, and 0.4504 × 10^–3^ (g/msPa), respectively. These data revert to (a) film thickness. (b) The ability of nanoparticles to fill the pores between the HPMC films structure. (c) HPMC diffusion with different nanoparticles and form homogenized structure^37^. The value of the film thickness (x) of HMPC control was 0.164 µm. The values of thickness of edible films reinforced with AgNPs and TiO_2_NPs were 0. 1855 and 0.1455 µm, respectively. These results are in agreement with those obtained by Jiménez et al^37^, Silva-Weiss et al^38^, Ahmadi et al^39^, Osorio et al^40^ and Sievens-Figueroa et al^41^.

**Table 6:** Water vapor permeability of bio-composite film reinforced with nano-particles after 24 h.

Time (min)	Weight gain (g)						Thickness (µm) (x)	WVP (× 10^–3^ g/msPa)
	0	60	120	180	240	1440		
HMPC	0	0.01605 ± 0.001	0.04705 ± 0.01	0.08315 ± 0.02	0.12415 ± 0.02	0.75955 ± 0.03	0.164	0.5076
HMPC-Ag NPs	0	0.0167 ± 0.02	0.0405 ± 0.03	0.06335 ± 0.03	0.09275 ± 0.04	0.62015 ± 0.08	0.1855	0.4596
HMPC-TiO_2_ NPs	0	0.0194 ± 0.01	0.04675 ± 0.02	0.07735 ± 0.03	0.112 ± 0.03	0.68645 ± 0.1	0.1455	0.4504

Values were presented as mean ± standard deviation (SD).HMPC: hydroxyl propyl methyl cellulose film, HMPC-AgNPs: HMPC film reinforced with silver Nanoparticles, and HMPC-TiO_2_NPs: HMPC film reinforced with titanium oxide nanoparticles.

#### Transparency

Table 7 shows the transparency of the bio-composite film based on HPMC film reinforced with nanoparticle of Ag NPs and TiO_2_-NPs compared to HPMC only. It could be seen that the visible light peak (VL) at different wavelengths 395, 430 and 550 nm ranged from 45 to 63% for HPMC film reinforced with Ag NPs and TiO_2_-NPs nanoparticle films, which it ranged from 58 to 73% for HPMC control. That is due to the difference in films reinforced with nanoparticles color^42^.

**Table 7:** Transparency of bio-composite film based on HPMC reinforced with nanoparticles (Ag- NPs and TiO_2_-NPs).

Properties	HMPC-Control			HMPC-AgNPs			HMPC-TiO_2_ NPs		
Transparency value	550 nm %VL	430 nm %BL	395 nm %PL	550 nm %VL	430 nm %BL	395 nm %PL	550 nm %VL	430 nm %BL	395 nm %PL
	73	58	69	58	57	55	63	45	51

*PL* Purple light Peak wavelength: 395 nm, *BL* Blue light Peak wavelength: 460 nm and *VL* Visible light Peak wavelength: 550 nm. *HMPC* hydroxyl propyl methyl cellulose film, *HMPC-AgNPs* HMPC film reinforced with silver Nanoparticles, and *HMPC-TiO*_*2*_*NPs* HMPC film reinforced with titanium oxide nanoparticles.

## Conclusion

The bio-composite film based on Hydroxypropyl methylcellulose (HPMC) was prepared with concentrations of AgNPs and TiO_2-_ NPs by casting method. The results have shown that some properties of the bio-composite were affected by AgNPs and TiO_2-_ NPs content. AgNPs and TiO_2-_ NPs caused an improvement in the WVP of the bio-composites. Transparency in control film was more value than HMPC film reinforced different nanoparticles samples. The mechanical resistance of the film increased after the formation of composite with AgNPs and TiO_2_ NPs. However, addition of nanoparticles resulted in increased values of elongation at break. Bio-composite films reinforced with AgNPs and TiO_2-_NPs incorporated demonstrated strong antibacterial activity against both the Gram-positive and Gram-negative food borne pathogenic bacteria. The added AgNPs and TiO_2-_ NPs to HMPC film, can be used as novel active food packaging materials to prolong the shelf-life of food during storage. However, further studies are needed to determine the potential toxicity of nano-materials released from the biopolymer matrix and their migration to food.

## Data Availability

The datasets used and/or analyzed during the current study available from the corresponding author on reasonable request.

## List of symbols

A: Cross sectional area of the film sample
AgNPs: Silver nanoparticles
C: Slope of the weight gain of the dish
D: Crystallite size
E: Elongation
EM: Young’s elastic modulus
F_max_: Max load needed to pull the sample apart
HPMC: Hydroxypropyl methylcellulose
l_max_: Film elongation at that moment of rupture and
l_o_: Initial grip length of the sample
MC: Moisture content
ROS: Reactive oxygen species
SM: Soluble matter
TiO_2_-NPs: Titanium oxide nanoparticles
TS: Tensile strength
WVP: Water vapor permeability
X: Film thickness
ΔP: Water vapor pressure differential across the film
λ: X-ray wavelength
β: Full width at have maximum of the peak
θ: Central angle of the diffraction peak
